# Continuous Information Monitoring in Software Startups

**DOI:** 10.1007/978-3-030-58858-8_29

**Published:** 2020-08-18

**Authors:** Usman Rafiq, Xiaofeng Wang

**Affiliations:** 6 IT University of Copenhagen, Copenhagen, Denmark; 7 University of British Columbia, Vancouver, BC Canada; Faculty of Computer Science, Free University of Bozen-Bolzano, Bolzano, Italy

**Keywords:** Software startups, Information monitoring, Metric monitoring

## Abstract

Software startups are central nowadays and considered primary drivers of economy and innovation. Lean and agile approaches are suggested for software startups to continuously build and validate the product. Thereby, they need to balance between the speed to deliver product and the quality of the product. It further urges startups to continuously monitor the versatile information, adjust their directions, and keep the bird’s-eye view. However, the preliminary literature review highlights that software startups, especially at the early stages, are not even aware of the need for information monitoring. This research project aims to identify how software startups decide what information needs to be monitored. The research plan proposes to utilize multiple case-study and surveys as data collection methods while grounded theory and factor analysis as data analysis procedures. Overall, both qualitative and quantitative research methods are expected to be implemented. The prospective research results encompass a framework to decide what information needs to be continuously monitored.

## Introduction

Startups have peculiar characteristics and are human organizations aiming to innovate and grow under extreme conditions of uncertainty [6]. They strive to develop and offer new services or products. A rough estimate indicates that about 100 million new startups are created each year around the world [3]. Among them, there is a large pool of software startups. The venture capital group of IBM [17] states that software-based startups are central nowadays among the pool and considered primary drivers of economy and innovation. Success stories of many existing and successful startups like Facebook, Instagram, Dropbox, Airbnb and etc. are primarily contributing towards this radical uplift. Despite this latest trend, very little is known about failures. The statistics given in [4] show about 98% of startups that aim to present new products end up with failure. Whatsoever we may think of a startup failure reason, the statistics still remain alarming for all definitions of failure. For instance, Nobel and Carmen [5] reported that 90–95% of startups fail in the conception phase, 70–80% fail to see a return on projection, and 30–40% fail by losing everything.

Software startups share a lot with other types of startups; however, they are also required to cope with the frequently changing technological wave [7]. Time pressure is also critical when a software startup is at an early stage [11]. A software startup is at an early stage when it struggles to conceptualize the idea and float it to the market for the first time [11]. These all factors urge software startups to balance between the speed and the quality of the product [14]. Pantiuchina et al. [14] also indicate that software startups combine agile and lean startup approaches to build the product. Similar results are reported in [2, 8]. While agile helps to manage product development, the lean startup approach helps to continuously validate product ideas with the help of potential customer collaboration [8]. These pieces of evidence suggest that “moving fast” is a compulsion for software startups. While doing so, a software startup continuously communicates with various stakeholders including the team itself to meet both short and long-term goals [2].

Gislaine Camila et al. [2] highlight that communication, direct or indirect, brings a lot of changes in the original idea and startups need to continuously adjust their product and business models to conform to the requirements of their customers. The team needs to continuously communicate and discuss the day-day operations and also look at past decisions to make better ones in the future [2]. The early-stage startup teams are usually small, and decisions are taken by the team as a whole, involving co-founders, playing multiple roles in startup [13].

There is still uncertainty, however, how software startups, especially, at early stages, continuously monitor the versatile information and keep the bird’s-eye view. The study [12] reveals that the majority of the startups, especially at early stages, are not even aware of the dire need for information measurements. Whilst the body of literature on software startups is growing, however, most studies are conducted on software development methodologies [7, 16]. A search of the literature only revealed limited studies that discussed a few aspects of the information monitoring in software startups. However, the studies we found, such as [12, 13], only discuss the importance of monitoring metrics and benefits of measuring it. Despite the listed benefits, software startups are also not using consolidated information measurement tools [3]. Therefore, software startups must utilize information monitoring to deliver better quality products, maintain good momentum, and stay competitive, while moving fast [2, 12]. The term ‘information’ refers to the sum of data and associated meaning, which holds further power to generate knowledge [23].

## Aim and Objectives

The aims of this study are to understand what startups are currently doing regarding information monitoring and investigate how information monitoring could serve them better. Our initial literature review highlights the lack of studies on the utilization of information monitoring in startups. Regarding the second aim, we did not find how software startups decide what key information they shall monitor. Both qualitative and quantitative methods will be utilized to meet the research aims. The resultant framework will ease software startups to decide what is really important for them to monitor according to their needs. This will further enable them to filter the flood of available information to grow and take corrective action when required.

## Research Questions

The main research question that we plan to answer is:

- **RQ1: How to identify what information a software startup needs to monitor continuously?**

This main research question is decomposed into the following aspects of continuous information monitoring in software startups:

- **RQ1.1:** What information is available for startups to monitor?
- **RQ1.2:** What are the enablers and inhibitors of information monitoring in software startups?
- **RQ1.3:** How do software startups decide what information they need to monitor continuously?

The initial literature review reveals a lot of results about the large pool of information on measurements in software startups i.e. RQ 1.1. On the other hand, it clearly lacks on how the startups decided what is really important for them and what makes them do so.

- **Expected Results:**
  - Enablers and inhibitors of information monitoring in software startups.
  - A framework enabling a particular software startup to identify the key information that needs to be monitored continuously.
  - A Goal Question Metric for software startups based on the prospective framework.

We also foresee that the information monitoring process inside software startups would be iterative, flexible, and robust i.e. continuous. This indicates that once the key information is identified and monitored then the startup may decide to look for the additional information again in the next iteration. As a result, a modification in the required key information will occur. According to [19], the process of learning and discovery brings changes in a startup and takes it to the different life-cycle stages. This process goes in parallel with the product development process [19]. A startup is also divided into different stages according to the product development state. These stages are concept in-development, working prototype, functional product with limited users, functional product with high growth, and mature product [19]. When a startup moves to the next stage as a result of learning then there seems to be the need to monitor additional or different perspectives. Accordingly, this research proposes to monitor the key information continuously i.e. across all life-cycle stages of a startup.

## Related Work

The key studies were identified using multiple search strings. The initial search string was based on the keywords, or their synonyms, used in the research questions. However, reading the search results revealed additional keywords that were later added to the list. The sources selected for this search were Scopus, Google Scholar, and IEEE Xplore. The search was performed in the title, abstract, and keywords fields. Likewise, search strings were developed by merging keywords information monitoring and startup. We did not particularly look for information monitoring in software startups as we were expecting a few results on the topic. Therefore, all the articles ranging from general startup organizations to software startups, in particular, were also undertaken.

The search results were first examined using the titles of the articles. However, in some of the cases, the abstracts were also used to know whether the particular article answers any of the research questions. Articles were also excluded if the word startup or start-up did not refer to the startup organization. Our search stems up from two sources i.e. information monitoring and startups. For information monitoring, we used keywords like information monitor, metric monitor, knowledge monitor, performance analysis, performance monitor, knowledge acquisition, performance indication, key performance indication, key performance data, monitor, and dashboard. Similarly, for the startup, we used startup, start-up, software startup, and software start-up.

### Findings

The literature review findings are classified and discussed according to the research questions. It is also found that the results were mainly discussing a few or none of the aspects of information monitoring.

Identification of key information that needs to be monitored is the very first and crucial challenge of this research. The initial literature review revealed very few published studies that discussed some aspects of information identification. The found studies provide a long list of recommended measurements and metrics in software startups while what is not clear is how software startups decide the most relevant information that needs to be monitored continuously. In the same vein, we did not find factors that motivate or restrict startups to monitor information. For instance, recently Kamulegeya et al. [12], studied 19 nascent software startups of the East-African region and concluded that software startups are measuring or wish to measure relevant information and also aware of the benefits of measurements. A similar conclusion has been echoed by [13] while stressing the use of data in the form of metrics to make better decisions. While performing a multi-vocal literature review and considering the practitioner’s opinions, available on the web, Kemell et al. [13] produced more than 100 different metrics. However, these metrics are not validated with the software startups and we also believe that a major portion of this list of metrics is applicable for more mature software startups. In contrast to Kemell et al. [13], Kamulegeya et al. [12] also studied software startups, particularly at early stages, and also produced a list of metrics that is important for software startups. The list seems comprehensive and well-classified. However, we found a limited similarity in the results of [12, 13].

Kamulegeya et al. [12] based their study on the measurements in large and established software companies [18] and compared their results with a practitioner’s book, known as “Lean Analytics” [10] that motivates software entrepreneurs to start measuring. While highlighting the paucity of studies on measurements in software startups, authors [12] conclude that software startups are using several measurements. The word metric and measurements are used interchangeably in this article. They classify the metric, being measured, or wish to be measured in software startups, into five categories. These categories include business-oriented, product-oriented, organizational performance-oriented, project-oriented and design-oriented metrics. This categorization is originally discussed in the study of measurements in large organizations [18]. Overall, 28 metrics were found belonging to one of these categories. It is interesting to relate that most software startups (17 out of 19) were using at least one of the business-oriented metrics while no one was using or wish to use design-oriented metrics. Likewise, what stands out in [12] is that the majority of startups, 12 out of 19, were not satisfied with what they were measuring and believed that they shall measure several aspects of software startups more adequately.

Based on the results discussed in [12], a multiple case-study report, we present categories and a list of associated key information that software startups, particularly at early stages, are measuring or wish to measure. The first category is related to business and associated key metrics include customer analytics (number of people using platform, customer behaviour), product delivery process time estimation, rate of customer/partner acquisition/growing customer base, revenue growth/generated revenue/activities that generate revenue, using a telemetry tool, tracking market indicators/market events, set and review business targets, product awareness/customer interest, using market as a benchmark, customer feedback measurement, reaching key business milestones (patents, tax registration, incorporation). The second category, product-oriented information, covers product/feature usage, production process time estimation, system reliability, ability to build a complete product, feedback from friends about product features (peer endorsement), product maintenance/support, and comparing product versions (added features). The third category, organizational performance measures, set and evaluate key performance indicators, time-based task setting, tracking and review for progress of project and time-based project performance appraisal. The fourth category, project metrics, monitors monetary value of time spent on task/activity, set and evaluate tasks, activity completion time, process adherence by the team, tools usage by team, product maintenance/support, documenting, and reviewing activities for progress. Lastly, not a single startup was found measuring design metrics.

Together, these studies [12, 13] indicate that data is worthwhile and provides deep insights if measured properly. Alongside, in another study, Kamulegeya et al. [13] interestingly, glimpsed a little about the Goal Question Metric (GQM) approach and concluded that GQM can also be used to identify the data required to be measured during business operations. While highlighting the limitations of GQM in only revealing productivity and quality metrics, they concluded that software startups are also required to look at other aspects such as business and technical activities. On that basis, we propose to build a GQM for software startups, including but not limited to product quality and team productivity.

A significant contribution to the use of data in software startups was introduced by Croll and Yoskovitz [10] in their book on lean analytics. The book explained to grow the startup faster and better by using measurements and metrics. Croll and Yoskovitz [10] categorized software startups according to six different business models and the relevant metrics were defined and associated with each kind of model. The business models include e-commerce, Software as a Service (SaaS), free mobile app, two-sided marketplaces, media sites, and startups providing user-generated content. Startups were also classified using the stages of software startups when startups sell to the enterprise. The stages referred to as empathy, stickiness, virality, revenue, and scale. The book [10] further defined the criteria to make sure that the startup is choosing the right metrics. Here, metrics were classified into five categories (qualitative versus quantitative metrics, vanity versus actionable metrics, exploratory versus reporting metrics, leading versus lagging metrics, and lastly correlated versus causal metrics). On the other hand, in contrast to the practitioner’s opinion [6], Kamulegeya et al. [12] claimed that the startup models which are listed in [10] are not sufficient to cover the types of startups in the market. They also pointed out that the metric provided by the lean analytics framework [10] specifically targets established software startups in developed economies and does not apply to emerging and nascent ecosystems.

## Research Methods

The research design consists of both qualitative and quantitative research methods. This research study aims to have a thorough understanding of what startups are currently doing regarding information monitoring. It highlights the nature of the study i.e. exploratory nature, particularly at initial stages; therefore, multiple case study method [22] is considered as the appropriate research method. Moreover, we also consider multiple case study approach as suitable due to the startup context and limited existing literature on the topic. The decision of using a multiple-case study approach is inspired by the recommendations provided by Easterbrook et al. [21]. A major advantage of using a multiple-case study is that it brings greater generalizability and validity control. Consequently, it is much easier to conclude strong and reliable results by investigating multiple cases.

To select the sample for our study, we propose to stratify cases according to the product type or business model and product development stages. Six classifications of software startups are indicated in [10] based on the product type and business models. Overall, therefore, we expect a combination of semi-structured interviews and observations to explore the cases for further analysis. Accordingly, interpretivist approach [20] is found suitable for this investigation based on the focus of research, nature of startups, and the use of interviews and observations as data collection methods. Going in the same vein, survey research is also being proposed to bring methodological triangulation for the initial findings. We also propose to conduct multiple interview sessions with startup representatives by involving multiple researchers to control validity threats.

Similarly, we plan to utilize both inductive and deductive data analysis approaches to analyze the data. In the inductive analysis, Grounded Theory (GT) [20] is proposed to analyze the qualitative data. It has been suggested that GT should be considered when there are no existing theories, or the explanation of a phenomenon is inadequate [20]. On the other hand, for deductive analysis, we will explore the relationships between variables using factor analysis [20].

## Conclusions

Software startups are different from other software development organizations, as they need to handle business aspects as well along with the technical challenges, in a very limited time. They are required to monitor information continuously to make better decisions in the future, adjust their directions in finding the right product idea, maintain the momentum and balance between speed and quality. While the literature on software startups is growing, there is still uncertainty, however, how software startups need to monitor the key information continuously. Likewise, existing software engineering tools and practices are considered heavy for software startups. Therefore, the current research project aims to address the challenge of understanding how software startups decide what information needs to be monitored continuously. To conduct the study, we plan to employ multiple case studies using interviews and observations, and survey. Similarly, to analyze the data, we plan to implement grounded theory and factor analysis approaches. Taken together, the research will take an interpretivist approach to execute the work.
